# CGI-58 Protein Acts as a Positive Regulator of Triacylglycerol Accumulation in *Phaeodactylum tricornutum*

**DOI:** 10.4014/jmb.2209.09029

**Published:** 2022-12-01

**Authors:** Qin Shu, Yufang Pan, Hanhua Hu

**Affiliations:** 1 Key Laboratory of Algal Biology, Institute of Hydrobiology, Chinese Academy of Sciences, Wuhan 430072, P.R. China; 2 University of Chinese Academy of Sciences, Beijing 100049, P.R. China

**Keywords:** CGI-58, diatom, *Phaeodactylum tricornutum*, triacylglycerol, eicosapentaenoic acid, lipid metabolism

## Abstract

Comparative gene identification-58 (CGI-58) is an activating protein of triacylglycerol (TAG) lipase. It has a variety of catalytic activities whereby it may play different roles in diverse organisms. In this study, a homolog of CGI-58 in *Phaeodactylum tricornutum* (PtCGI-58) was identified. PtCGI-58 was localized in mitochondria by GFP fusion protein analysis, which is different from the reported subcellular localization of CGI-58 in animals and plants. Respectively, *PtCGI-58* overexpression resulted in increased neutral lipid content and TAG accumulation by 42-46% and 21-32%. Likewise, it also increased the relative content of eicosapentaenoic acid (EPA), and in particular, the EPA content in TAGs almost doubled. Transcript levels of genes involved in de novo fatty acid synthesis and mitochondrial β-oxidation were significantly upregulated in *PtCGI-58* overexpression strains compared with wild-type cells. Our findings suggest that PtCGI-58 may mediate the breakdown of lipids in mitochondria and the recycling of acyl chains derived from mitochondrial β-oxidation into TAG biosynthesis. Moreover, this study potentially illuminates new functions for CGI-58 in lipid homeostasis and provides a strategy to enrich EPA in algal TAGs.

## Introduction

Diatoms are unicellular, photosynthetic eukaryotic microalgae that are responsible for 40% of marine biomass production each year, and at least 20% of the annual primary productivity of the Earth’s biosphere [1, 2]. The end products of photosynthesis are partly stored as triacylglycerol (TAG) in lipid droplets under certain conditions [3]. *Phaeodactylum tricornutum* is considered a model species for diatom research, especially for lipid research [4]. TAG degradation is a crucial process in diatoms for cellular survival and growth, and under fluctuating environmental conditions, it enables cells to rapidly mobilize storage lipids [5]. The initial step of this degradation of storage TAGs is generally catalyzed by lipases (E.C. 3.1.1.3).

Triacylglycerol lipase, also known as triacylglycerol acylhydrolase, is widely present in animals, plants, and microorganisms and plays a vital role in regulating lipid catabolism [6]. Comparative gene identification-58 (CGI-58), also known as α/β-hydrolase domain-containing protein 5 (ABHD5), belongs to the lipase subfamily [7]. CGI-58 is a key regulator of lipid metabolism and its mutation causes various neutral lipid disorders [8]. CGI-58 is localized on the surface of lipid droplets (LDs) in mammals [9, 10], and co-regulates adipose triglyceride lipase (ATGL) activity through the binding-dissociation interaction with LD proteins, thus regulating the decomposition efficiency of TAG in LDs [8]. In mice, deficiency of CGI-58 results in systemic multi-tissue TAG accumulation [11]. In *Arabidopsis thaliana*, earlier studies showed that CGI-58 protein had obvious cytoplasmic localization characteristics [12], but it was later found to mainly aggregate on the surface of peroxisome through interaction with the peroxisomal ABC-transporter1 (PXA1) to regulate lipid metabolism homeostasis [13]. The disruption of the *CGI-58* gene in *Arabidopsis* results in a 10-fold increase of lipid levels in mature leaves [12]. Knockdown of the homolog of CGI-58 (Thaps3_264297) in *Thalassiosira pseudonana* also resulted in increased lipid accumulation without affecting growth [14], which further validates the importance of CGI-58 for lipid metabolism.

Although the localization of CGI-58 in animals and plants is different, its role as an ATGL activator in lipid metabolism is clear, and it possesses various catalytic activities in different organisms. *Saccharomyces cerevisiae* and mouse CGI-58 showed lysophosphatidic acid acyltransferase (LPAAT) activity when expressed in *Escherichia coli* [15, 16], and CGI-58 homologs from *Arabidopsis* and *T. pseudonana* had additional lipase and phospholipase activities [14, 17]. However, McMahon *et al*. [18] attributed the LPAAT activity to the bacterial contaminant of plsC. In this study, a *P. tricornutum* CGI-58 (PtCGI-58) homolog was identified and fused with enhanced green fluorescent protein (eGFP) to determine its subcellular localization. The effect of PtCGI-58 on lipid metabolism was elucidated by constructing *PtCGI-58* overexpression lines. Our results suggest that the localization of PtCGI-58 differs from that reported in animals and plants, and overexpression of *PtCGI-58* promoted de novo fatty acid synthesis and mitochondrial β-oxidation, thereby enhancing the accumulation of TAG under nitrogen- deficient conditions.

## Materials and Methods

### Growth Conditions

Axenic cultures of *P. tricornutum* Bohlin (CCMP2561) (from the culture collection of the Provasoli-Guillard National Center for Culture of Marine Phytoplankton, Bigelow Laboratory for Ocean Sciences, USA) and transformants were cultivated at 22°C under continuous illumination of 100 μmol photons/m^2^/s without shaking in artificial seawater enriched with f/2 nutrients (nitrate concentration was reduced to 500 μM) [19]. The initial cell density of the batch culture was 2.5 × 10^5^/ml, and the growth of triplicate cultures of *P. tricornutum* was checked using a Malassez chamber (0.01 μl for a rectangle). The nitrate concentration was evaluated using a spectrophotometer at 220 nm [20].

### Phylogenetic Analysis, Vector Construction and Transformation

According to Ensembl database annotations and expressed sequence tag libraries [21], the full-length coding sequence of PtCGI-58 (Phatr3_J54974) was identified with a length of 1,485 bp (494 aa). A maximum likelihood (ML) analysis was performed using the PHYML algorithm [22] for CGI-58 homologs in *P. tricornutum* and closely related species with S. cerevisiae as the out-group to root the tree.

To generate *PtCGI-58* overexpression strains of *P. tricornutum*, the entire coding region was amplified using the primers CGI-58-BamHI-Fw and CGI-58-OE-Rev by PCR and then inserted into the plasmid pPha-T1 [23] between the XbaI and BamHI sites to obtain the pPhaT1-CGI-58-OE expression vector. For the subcellular localization analysis of PtCGI-58, the pPhaT1-CGI-58-eGFP construct was generated to express C-terminal eGFP fusion proteins in *P. tricornutum* cells. Primers CGI-58-BamHI-Fw and CGI-58-eGFP-Rev were used to amplify the full-length *PtCGI-58*, and the resulting open reading frame was inserted into pPhaT1-linker-eGFP (between the BamHI and XbaI sites), which was obtained by inserting the linker sequence (GGACCTAGGGGA GGAGGAGGAGGA) and the eGFP coding sequence [24] into the XbaI and HindIII sites of pPha-T1. The hydrophilic and highly immunogenic peptides from positions 274 to 486 in the PtCGI-58 protein were selected to be expressed in *E. coli*. The selected DNA fragment (820-1458 bp of *PtCGI-58* gene) was amplified by PCR using primers CGI-58-820BamHI-Fw and CGI-58-1458SacI-Rev, and was then inserted into the SacI and BamHI sites of pET28a vector to obtain the pET28a-PtCGI-58 (820-1458) construct for PtCGI-58-specific antibody preparation. Primers used for vector construction are shown in the Supplemental Table.

The linearized (by NdeI) expression vector was transferred into the wild-type (WT) *P. tricornutum* by electroporation following the method of Zhang and Hu [24] and transformants were selected on a solid-medium plate containing zeocin resistance (100 μg/ml) at 22°C and continuous light. To observe the fluorescent signals, pPhaT1-CGI-58-eGFP transformants and the WT cells were incubated in 190 mM MitoTracker Orange (Invitrogen, USA) for mitochondrial staining or in 0.1 μg/ml boron-dipyrromethene (BODIPY 505/515, Invitrogen) dyes for LD staining (protected from light). Cells were washed once with f/2 medium after staining and then observed using a Leica TCS SP8 laser scanning confocal microscope. The excitation light wavelength for eGFP fluorescence, chloroplast autofluorescence, and BODIPY fluorescence was 488 nm and the detection wavelengths were 500-550 nm, 630-690 nm, and 500-530 nm, respectively. The MitoTracker Orange fluorescence was excited at 552 nm and detected at a bandwidth of 560–590 nm.

### Real-Time Quantitative PCR (RT-qPCR) and Western Blot

To examine the relative expression levels of *PtCGI-58* mRNA in WT and transformants, cells cultured for 4, 6 and 8 days were harvested by centrifugation at 3,000 ×*g* for 10 min and used for RNA extraction with Trizol reagent (TaKaRa, China). The first-strand cDNA reverse transcribed from RNA according to the HiScript III 1st Strand cDNA Synthesis Kit (Vazyme, R312-01/02, China) was used as template for RT-qPCR using LightCycler 480 SYBR Green I Master (Roche, Germany) and a LightCycler 480 Real-Time PCR System (Roche). The primers used for RT-qPCR were shown in the Supplemental Table. The relative expression quantification of the target gene was calculated using *histone* H4 as the endogenous control gene [25] according to the 2^−ΔΔCt^ method [26].

Cells grown for 3 days were harvested by centrifugation for protein extraction using Western/IP lysis buffer (Beyotime, China), and the protein concentration was determined with the BCA Protein Concentration Assay Kit (Beyotime). The protein samples were isolated on a 12% SDS-PAGE gel before being transferred to PVDF membrane and hybridized with PtCGI-58 polyclonal antibody prior to HRP-labeled goat anti-rabbit IgG secondary antibody. After extensive washing, the blots were developed by ECL (Millipore, USA), and chemiluminescence was captured on an ImageQuant LAS 4000 mini apparatus (GE Healthcare Life Sciences, UK).

### Neutral Lipid and TAG Content Analysis

Relative neutral lipid content was determined by fluorescence spectroscopy using the Nile red dye (Sigma-Aldrich, USA) [4]. Briefly, equal numbers of cells (6 ×10^6^ cells) were harvested and resuspended to 3 ml, and then 30 µl (100 µg/ml) Nile red staining solution was added at 37°C for 30 min. The excitation wavelength of the sample was set at 531 nm and the fluorescence emission wavelength was 572 nm in a Perkin-Elmer LS55 fluorescence spectrometer.

For TAG content analysis, the same amount of algal cells grown to day 6, 8, and 10 were collected by centrifugation (4000 ×*g*, 4°C, 10 min), and total lipids were extracted by chloroform/methanol (1:1 by volume) [27]. TAGs were separated by one-dimensional thin-layer chromatography (TLC) [28] in hexane:diethyl ether:acetic acid (70:30:1 by volume) on silica gel plates. Bands were visualized by exposure to iodine vapor at 37°C for 15 min using triolein as standard (Sigma-Aldrich), and relative quantitative analysis of TAG spots was performed with ImageJ.

### Fatty Acid Profile and TAG Composition Analysis

For the analysis of fatty acid profile and TAG composition, cells cultured for 14 days were collected by centrifugation and then freeze-dried. Total lipids were extracted from 100 mg dry powder and then dissolved in 1 ml chloroform, 200 μl of which was used for methyl esterification and fatty acid profile analysis by gas chromatography (TRACE GC, Thermo Scientific, Italy) with a capillary column (60 m × 0.25 mm) (DB-23, J&W Scientific, USA). Fatty acids were identified by comparison of their retention times with those of standards (Sigma) and quantified using C_17:0_ as internal standard. The remaining total lipids were analyzed by liquid chromatography-tandem mass spectrometry (LC-MS/MS) for TAG composition [29].

### Statistical Analysis

Data were presented as mean and standard deviations from the duplicates. Statistical analyses of the data were performed with *t*-test, and *p* < 0.05 was considered statistically significant.

## Results and Discussion

### Sequence Analysis and Subcellular Localization of PtCGI-58

**Fig. 1:**
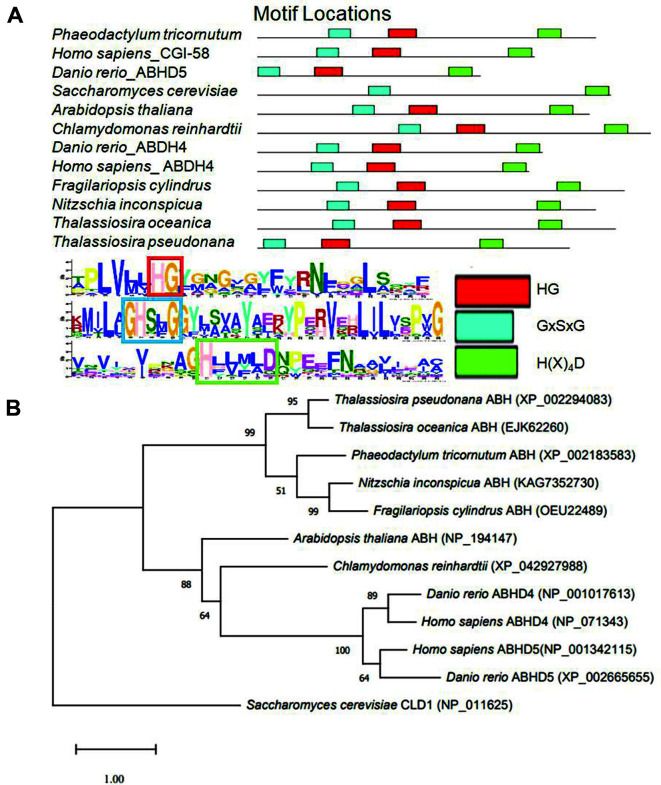
Amino acid sequence alignment (A) and phylogenetic relationships (B) of CGI-58 proteins from different species. HG: His-Gly lipid-binding domain, GxSxG: catalytic serine lipase motif, H(X)4D: acyltransferase motif. Numbers above branches represent the support values. The scale bar represents the evolutionary distances.

PtCGI-58 (Phatr3_J54974) was identified by BLAST search of the *P. tricornutum* protein database using human CGI-58 protein as a query sequence, which showed a 33% identity as a homolog of human CGI-58 protein. The N-terminal Trp-rich region of CGI-58 protein makes it a potent activator of ATGL involved in lipolysis [30]. The amino acid sequences of PtCGI-58 and CGI-58 homologous proteins from other species were analyzed by the MEME suite (https://meme-suite.org), revealing that PtCGI-58 and most of the CGI-58 homologs contained the lipid-binding site His-Gly dipeptide, the lipase motif GxSxG, and an acyltransferase H(X)_4_D active site located in the C-terminal region [7, 31, 32] (Fig. 1A). PtCGI-58 shared 50, 47, and 46% sequence identity with the CGI-58 homologs in diatoms *T. pseudonana*, *Nitzschia inconspicua*, and *Fragilariopsis cylindrus*, respectively. We constructed an ML tree to show the phylogenetic relationship between CGI-58 proteins in different species (Fig. 1B), and the five diatom CGI-58 proteins were clustered together, indicating an independent evolutionary origin.

**Fig. 2:**
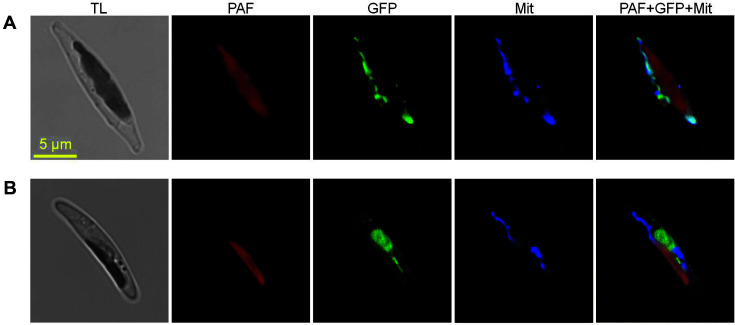
Fluorescent microscope images of cells expressing PtCGI-58-eGFP (A) or the empty vector (pPhaT1- linker-eGFP) (B). TL: transmitted light, PAF: plastid autofluorescence, GFP: GFP fluorescence, Mit: mitochondria stained by MitoTracker Orange, PAF+GFP+Mit: a merged image.

CGI-58 homologous protein has been reported to localize on the surface of LDs in mammals [9, 10] or in the cytoplasm (mainly aggregated on the surface of peroxisome) in *Arabidopsis* [12, 13]. PtCGI-58 was predicted to contain no signal peptide, including that of mitochondrial targeting sequence, by SignalP - 6.0 (https://services.healthtech.dtu.dk/) and iPSORT (https://ipsort.hgc.jp/). Prediction by TargetP (https://services.healthtech.dtu.dk/) and HECTAR (the heterokont subcellular localization targeting method) generated low probability scores (0.025594 and 0.1201 respectively) for the presence of mitochondrial signals. However, predictions by Loctree3, which were made by inference of localization information from experimentally annotated sequence homologs using PSI-BLAST, showed a mitochondrion-localization of PtCGI-58. To observe the subcellular localization of PtCGI-58, the PtCGI-58:eGFP fusion protein was expressed in *P. tricornutum* cells. As shown in Fig. 2A, the eGFP fluorescence signal (green) was distributed around the red plastid fluorescence and showed parallel staining with a MitoTracker Orange (blue), which reflects a mitochondrial localization of PtCGI-58. By contrast, in the algal cells expressing eGFP alone, the green signal was present in the cytosol, distinct from the plastid autofluorescence (red) and stained mitochondria fluorescence (Fig. 2B). Different localizations of CGI-58 homologs in diatoms, plants, and mammals further indicate that they may have different evolutionary origins and functions.

### Effects of PtCGI-58 Overexpression on Cell Growth and TAG Content

**Fig. 3:**
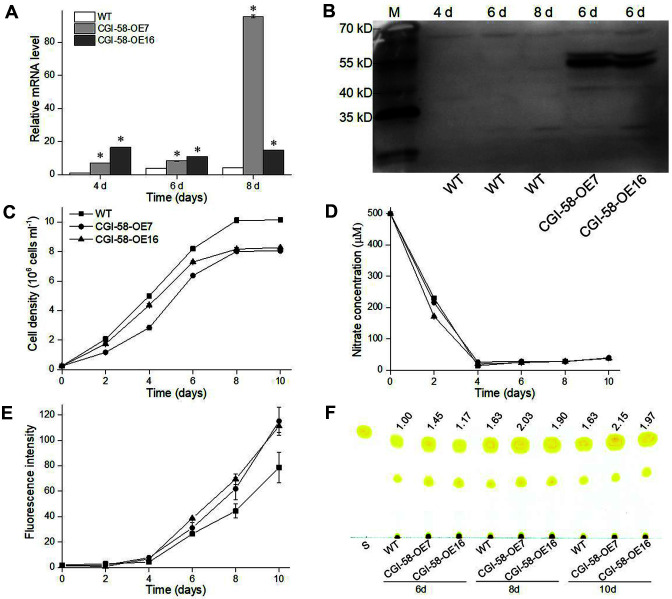
Effect of *PtCGI-58* overexpression on growth and neutral lipid accumulation. **A**, Relative mRNA level of *PtCGI-58* in two *PtCGI-58* overexpression (OE) lines and the wild-type (WT) at days 4, 6 and 8. **B**, Immunoblotting analysis of PtCGI-58 protein level in WT (days 4, 6 and 8) and the two *PtCGI-58* OE lines (day 6). **C**, Growth, **D**, Nitrate utilization, **E**, Accumulation of neutral lipid detected by Nile red assay. **F**, TAG content analyzed by thin-layer chromatography in WT and two OE lines (CGI-58-OE7 and CGI-58-OE16) grown in artificial seawater enriched with f/2 nutrients (nitrate concentration was reduced to 500 μM). Data are the average of three biological replicates with error bars indicating standard deviations. Asterisks indicate statistically significant differences from WT at *p* < 0.05 level.

To understand the role of PtCGI-58 in lipid metabolism, *PtCGI-58* overexpression (OE) strains of *P. tricornutum* were constructed. The relative mRNA levels of *PtCGI-58* in *PtCGI-58* OE strains and WT cells were analyzed on day 4, 6, and 8. From day 4 to 8, transcript levels of *PtCGI-58* were upregulated in the WT (Fig. 3A). The expression level of *PtCGI-58* was significantly higher in OE strains than that in WT cells (Fig. 3A), and immunoblotting showed that PtCGI-58 level in the two OE strains was higher at day 6 (Fig. 3B).

**Fig. 4:**
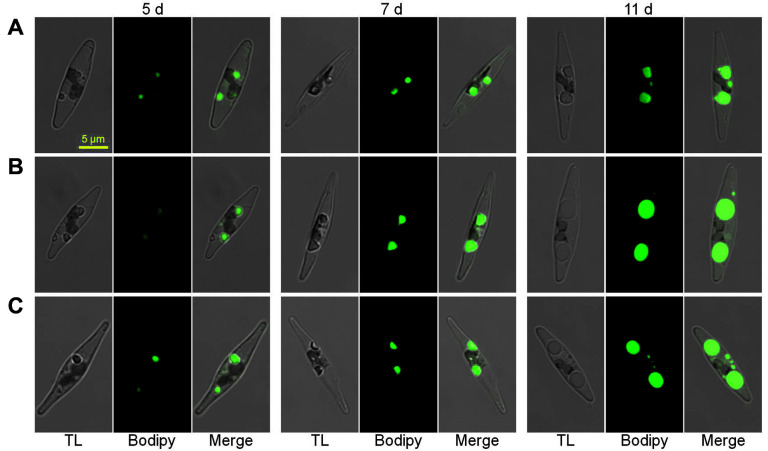
Oil accumulation levels monitored using BODIPY fluorescence in WT (A) and two *PtCGI-58* overexpression lines (B, CGI-58-OE7; C, CGI-58-OE16) and at days 5, 7 and 11. TL: transmitted light, Bodipy: stained oil droplet fluorescence, Merge: a merged image.

During the whole growth phase, the two *PtCGI-58* OE strains exhibited lower cell densities compared with the WT, and in particular, a decrease by 18-21% at day 10 (Fig. 3C). There was no difference in the utilization of nitrate between *PtCGI-58* OE strains and WT cells (Fig. 3D). Before nitrate was exhausted at day 4 (Fig. 3D), the neutral lipid was hardly detected by Nile red assay in both OE strains and WT cells (Fig. 3E). From day 6 to 10, much higher neutral lipid (Fig. 3E) and TAG (Fig. 3F) contents were achieved in the two *PtCGI-58* OE strains, and, in particular, neutral lipid contents increased by 42-46% at day 10 compared with what was observed in WT cells (Fig. 3E). Correspondingly, an increase of TAG content by 21-32% at day 10 was found in *PtCGI-58* OE strains relative to WT cells by TLC analysis (Fig. 3F). The intracellular oil droplets detected by BODIPY staining assay were bigger in size in *PtCGI-58* OE cells, and they all tended to enlarge from day 5 to 11 (Fig. 4). These results all suggest that PtCGI-58 plays an important role in the regulation of TAG accumulation.

Previous studies have shown that knockout or knockdown of *CGI-58* homolog results in an increased TAG accumulation in animals, plants, and *T. pseudonana* [11, 12, 14]. Overexpression of *CGI-58* in yeast showed a decrease in TAG level due to the coactivation of ATGL-like lipase by CGI-58 [32, 33]. Our results showed a distinct relationship between *CGI-58* expression and TAG accumulation. The WT neutral lipid started to accumulate at day 4 when nitrate was exhausted (Fig. 3D), while the transcript level of *PtCGI-58* was upregulated at days 6 and 8 compared with day 4 (Fig. 3A). The positive correlation between *PtCGI-58* expression and TAG accumulation was more significant in *PtCGI-58* OE strains, where more TAG accumulation and higher *PtCGI-58* expression levels were observed. These findings indicate that PtCGI-58 may have more diverse functions than the reported CGI-58 homologs.

### *PtCGI-58* Overexpression Increases EPA Accumulation in TAGs

**Fig. 5:**
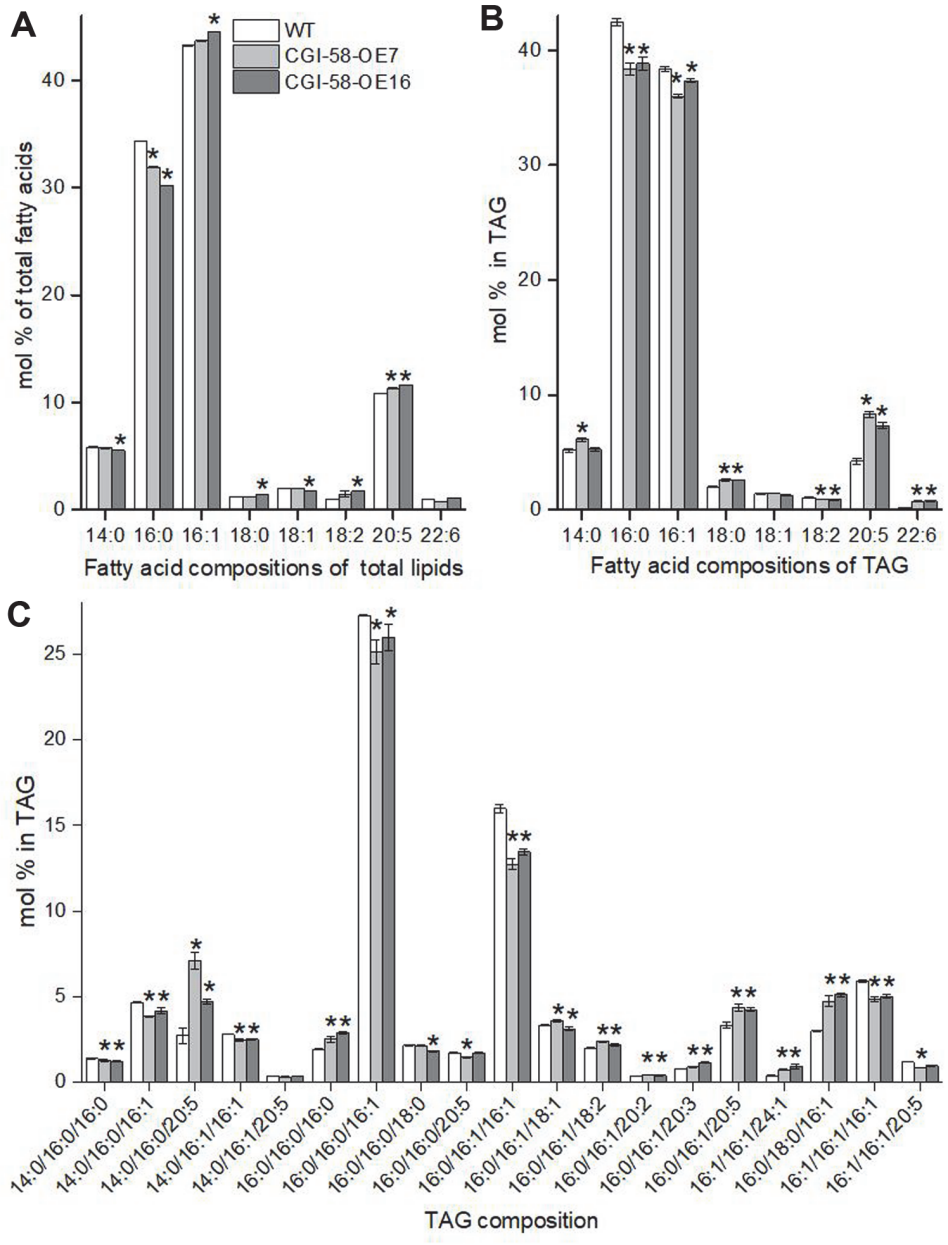
Comparative analysis of fatty acid composition in total lipid (A) and triacylglycerol (TAG) (B), and the TAG species (C) between WT and two overexpression strains (CGI-58-OE7 and CGI-58-OE16). Data are the average of three biological replicates with error bars indicating standard deviations. Asterisks indicate significant differences compared to WT at *p* < 0.05 level.

The fatty acid composition of total lipids and TAGs in *PtCGI-58* OE strains and WT were similarly dominated by C14:0, C_16:0_, C16:1, and C20:5 (eicosapentaenoic acid, EPA), and C_16:0_ and C16:1 accounted for more than 74% of total fatty acids in both total lipids and TAGs (Fig. 5). Overexpression of *PtCGI-58* resulted in a significant decrease in the relative content of C_16:0_ (*p* < 0.05) and an obvious increase of EPA (*p* < 0.05) in total lipid fatty acids. Meanwhile, there was hardly any difference in the contents of other fatty acids between the *PtCGI-58* OE lines and WT.

In terms of TAG fatty acid composition, the decrease of C_16:0_, C16:1, and C18:2 (*p* < 0.05), and the increase of C18:0, EPA, and C22:6 (docosahexaenoic acid, DHA) (*p* < 0.05) were observed in *PtCGI-58* OE strains, and in particular, the EPA content almost doubled (Fig. 5B). These results indicated that overexpression of *PtCGI-58* is beneficial to the improvement of long-chain polyunsaturated fatty acids (LC-PUFAs) in TAGs. There were 19 main TAG molecules in *P. tricornutum* based on LC-MS/MS analysis, among which TAG-48:1 (16:0/16:0/16:1), TAG-48:2 (16:0/16:1/16:1), TAG-46:1 (14:0/16:0/16:1), TAG-50:1 (16:0/18:0/16:1), TAG-48:3 (16:1/16:1/16:1), TAG-50:5 (14:0/16:0/20:5) and TAG-52:6 (16:0/16:1/20:5) were the most abundant (Fig. 5C). *PtCGI-58* overexpression resulted in a significant decrease in the TAG molecules 16:0/16:0/16:1 and 16:0/16:1/16:1, while effectuating a significant increase in the TAG molecules 14:0/16:0/20:5 and 16:0/16:1/20:5 (Fig. 5C). These TAG species should be the main molecules responsible for fatty acid changes in TAGs in *PtCGI-58* OE lines.

The composition of TAG molecular species was similar between wild-type and *cgi-58* loss-of-function mutants both in LDs and whole-leaf tissue in *A. thaliana* [12]. In *Thaps3_264297* knockdown strain of *T. pseudonana*, quantities of most of the fatty acid methyl esters species detected were increased, and in particular, levels of 16:0 and 18:2 fatty acids were significantly enriched after 40 h of silicon starvation [14]. Moreover, changes in fatty acid composition in TAGs were also found in some lipase knockdown strains in *P. tricornutum*. The outer envelope membrane (OEM)-localized lipase OmTGL-knockdown lines exhibited an increase of 68–70% in EPA content in TAGs [34], while SDP1 patatin-like lipase Tgl1 knockdown strains showed significantly increased relative content of C_16:0_ and decreased relative EPA content in TAGs [35]. These findings indicated that lipases with different subcellular locations may have various substrate specificities.

### Transcript Level Analysis Revealed *PtCGI-58* Overexpression Promotes De Novo Fatty Acid Synthesis and Mitochondrial β-Oxidation

**Fig. 6:**
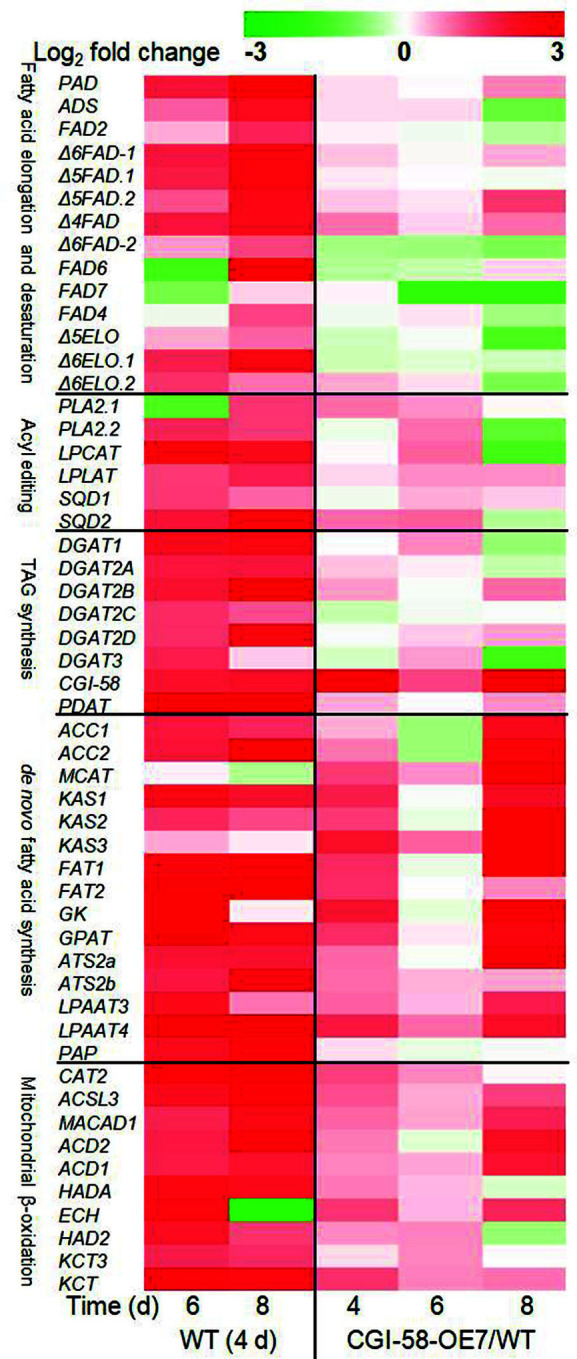
Transcription levels of genes related with lipid metabolism in WT (relative to day 4) and *PtCGI-58* overexpression line CGI-58-OE7 (relative to WT). WT: wild-type.

Based on the mitochondrial subcellular localization of PtCGI-58 and the phenotypes of *PtCGI-58* OE lines, transcription levels of genes related to TAG synthesis and mitochondrial β-oxidation were analyzed by RT-qPCR to elucidate how *PtCGI-58* overexpression promoted TAG accumulation. As shown in Fig. 6, transcript levels of most of the detected genes in WT cells were upregulated at days 6 and 8 compared with day 4, reflecting the enhancement of fatty acid de novo synthesis, elongation and desaturation, acyl editing, TAG synthesis, and mitochondrial β-oxidation, which was consistent with the significant accumulation of TAG in *P. tricornutum* cells at days 6 and 8.

In the *PtCGI-58* OE strains, transcription levels of genes related to fatty acid elongation and desaturation, acyl editing, and TAG synthesis were not significantly different from those in the WT, whereas most of the genes involved in fatty acid de novo synthesis and mitochondrial β-oxidation were significantly upregulated compared with the WT, especially at day 8 when the extensive TAG accumulation occurred. These trends emphasized the importance of de novo fatty acid synthesis and mitochondrial β-oxidation for promoting TAG accumulation in *PtCGI-58* OE cells. It has been reported that a functional mitochondrial fatty acid β-oxidation pathway exists in *P. tricornutum* and plays an important role in the degradation of TAG-derived fatty acids [36, 37]. We speculated that mitochondria-localized PtCGI-58 might mediate the breakdown of TAG and the recycling of acyl chains from mitochondrial β-oxidation, thereby resulting in increased TAG accumulation.

De novo synthesis of TAG mainly utilizes phosphatidylcholine (PC)-derived DAGs as substrates [38]. PC acyl editing and PC-DAG interconversion via PC:diacylglycerol cholinephosphotransferase (PDCT) control the majority of acyl flux through PC, thereby providing PUFAs for TAG synthesis in many plants [39]. It has been reported that recombinant plant CGI-58 purified from *E. coli* possesses hydrolase activities for PC in addition to TAG [17]. In addition, it was observed that the amount of phosphatidylglycerol (PG) was affected by the expression of CGI-58 in plants [12] and in *E. coli* [40]. Phospholipids PC and PG in *P. tricornutum* have a high proportion of EPA [41], and *PtCGI-58* overexpression may enhance the recycling of EPA released from the acyl-CoA pool following the degradation of PC and PG. This may partially explain the significant increase in the relative content of PUFAs, such as EPA and DHA, in TAGs of *PtCGI-58* overexpression strains compared with WT. It has been reported that overexpression of CGI-58 in S. cerevisiae showed an increase in the formation of phosphatidic acid [32]. In C2C12 cells, overexpression of CGI-58 exhibited significantly higher levels of endogenous PG and knockdown of CGI-58 reduced the endogenous PG level [42]. Therefore, further experiments are needed to study the relative phospholipid contents and their fatty acid compositions in *PtCGI-58* overexpression strains.

In summary, we identified a mitochondria-localized CGI-58 protein in *P. tricornutum*. Overexpression of *PtCGI-58* could significantly increase the content of TAG rich in PUFAs by promoting de novo fatty acid synthesis and mitochondrial β-oxidation. This study gives us a novel understanding of the function of PtCGI-58 protein in lipid metabolism and provides a new strategy to enrich EPA in algal TAG.

## Supplemental Materials

Supplementary data for this paper are available on-line only at http://jmb.or.kr.
