# Supplementary material for: CGI-58 Protein Acts as a Positive Regulator of Triacylglycerol Accumulation in Phaeodactylum tricornutum
Source: J Microbiol Biotechnol. 2022 Dec 1;33(2):242–50. doi: 10.4014/jmb.2209.09029 (PMC9998212; doi:10.4014/jmb.2209.09029)
Supplement: Supplementary file 1 [file jmb-33-2-242-supple.pdf]

J. Microbiol. Biotechnol.
https://doi.org/10.4014/jmb.2209.09029

Supplemental table. PCR primers used in this study.

Gene	Abbreviation	Protein ID (Phatr3_)	Forward (5'→3')	Reverse (5'→3')
**Vector construction (Restriction sites are underlined)**				
comparative gene identification-58	*CGI-58*	J54974	*CGI-58-BamHI-Fw*: CGggatccATGTTCGAAAGTGCTCCAAC	*CGI-58-OE-Rev*: GCtctagaCTATGACGCCACTTCAACAC
comparative gene identification-58	*CGI-58*	J54974		*CGI-58-eGFP-Rev*: GCtctagaCCATGACGCCACTTCAACAC
comparative gene identification-58	*CGI-58*	J54974	*CGI-58-820BamHI-Fw*: CGggatccCAGAAAGTTCTGGAAGAGCGC	*CGI-58-1458SacI-Rev*: CgagctcAGACATCAGTGCGCTGTGATT
**RT-qPCR**				
comparative gene identification-58	*CGI-58*	J54974	GTGTCCCGGAAGAGTCACAG	TCCGTACATCAGACGACCCT
palmitoyl-ACP desaturase	*PAD*	J9316	CCGCAAGATTACCTTCCCGA	GGGAGAGCTTCTTCCGTCAC
Δ9 acyl-CoA desaturase	*ADS*	J28797	GGCACAAGTCGCTTCCTACT	ATCGTAGGGGTGATCTCCGT
fatty acid desaturase 2	*FAD2*	J25769	GCTTTGCTCGTCCCCTACTT	GGCGTAGAAGGAGTTTCGCT
Δ6 fatty acid desaturase 1	*Δ6FAD-1*	J29488	TCGTCTTTTACTCGGACCGC	ACCTGGTGGTGCAGAAAGTC
Δ5 fatty acid desaturase 1	*Δ5FAD.1*	J46830	GGTCCTGTTTTCGTTGTCGC	CCACCGTAAGTGCAGGAAGT
Δ5 fatty acid desaturase 2	*Δ5FAD.2*	J22459	CTTTTTCGTTGCCGTGGTGT	TCCATACGTTCTTGCGTCCC
Δ4 fatty acid desaturase	*Δ4FAD*	J22510	GATTCGAATGCTTCCGCCAC	AGTGTTTGATGCGTTGTCGC
Δ6 fatty acid desaturase 2	*Δ6FAD-2*	EG02619	GACGACGAACAATGCCGAAC	ATAGCGCGTCCTTTTTCGGA
fatty acid desaturase 6	*FAD6*	J48423	TGGAACTGGGGGAAACACAC	GCCTCCGTATGCTTTCATGC
fatty acid desaturase 7	*FAD7*	J41570	CCGTGGTTGGTCCTGTCATT	TGCGATTGATCCACGTTCCA
fatty acid desaturase 4	*FAD4*	J5271	AGAAACCGAGACAACGGCTT	TTGTGCATTTCGCCCTTTGG
Δ5 fatty acid elongase	*Δ5ELO*	J34485	CATTGCCCTGCACAATCTCG	CCACAGCCCGAACTGATGTA
Δ6 fatty acid elongase 1	*Δ6ELO.1*	J22274	GCCTCTCGCCAATCTCCAAT	GCAGGAAGGAAAGTTGACGC
Δ6 fatty acid elongase 2	*Δ6ELO.2*	J20508	CGCAATTGACCCTTATCCGC	AGTCGTTGCAAGGCCAGAAT
phospholipase A2 1	*PLA2.1*	J39425	GACGGCTCCAGGATTGGAAA	TGCGGAATCGCGAAGATGTA
phospholipase A2 2	*PLA2.2*	J46193	TTGGGATACCGCCAAGGAAC	TGGTCGTACCATCTCCGAGT
lysophosphatidylcholine acyltransferase	*LPCAT*	J49702	TACGAGACTCGAAGCGATGC	TTGTACTTTCGGGCGGACTC
lysophospholipid acyltransferase	*LPLAT*	J20460	GGAAAACCTCGCGGCAAAAT	AAAACAACGGGCGTTGAACC
UDP-sulfoquinovose synthase	*SQD1*	J21201	GTACGGGTACGGAACTAGCG	ACTTGGTGGCATGGTAGACG
sulfoquinovosyltransferase	*SQD2*	J50356	GTTGGAGCTCGTCAACCTCA	GACAGGAGCAACCCACCAAT
acyl-CoA:diacylglycerol acyltransferase 1	*DGAT1*	J9794	GCCGGAAATACTTCAACGAA	AAAGCAAAATTGCACCAACC
acyl-CoA:diacylglycerol acyltransferase 2A	*DGAT2A*	J49462	GATCTGGCCTAAATCCGTCA	CGACGATGAGACGATCAAGA
acyl-CoA:diacylglycerol acyltransferase 2B	*DGAT2B*	J49544	GTCGACGTTTTCCATGGTCT	AAATGGCCGAGACAATCATC
acyl-CoA:diacylglycerol acyltransferase 2C	*DGAT2C*	J31662	GCCCTCCGACAATGTGTACT	CAACGGCCATGTGACTACAG
acyl-CoA:diacylglycerol acyltransferase 2D	*DGAT2D*	J43469	AATTTGTGTTCGCCGTTAGC	ACTGTCGCCGCTAGTGTTCT
acyl-CoA:diacylglycerol acyltransferase 3	*DGAT3*	J49708	TTGATGGGAAAAGGAGCATC	ATTTGCCGAAACAAGACACC
phospholipid:diacylglycerol acyltransferase	*PDAT*	J8860	TGGGTTGACGAGCATATTCA	CCCCATGTCGTCCATAAATC
acetyl-CoA carboxylase 1	*ACC1*	EG01955	ACCTTTACGGCAAGGAGGAT	ACGCTGAAGTATCCCCACAC
acetyl-CoA carboxylase 2	*ACC2*	55209	ATGATCAAGGCCAGTGAAGG	GTATTCGTCCGCCAACAACT
malonyl-CoA:ACP transacylase	*MCAT*	37652	GTGAGGCCGTCAATGAACTC	ACCTTTTCCAAGGAGGCAAC
3-ketoacyl-ACP synthase 1	*KAS1*	18940	TGCTGGAAGTCACGCTATTG	CGACAAAACCCAGCCATACT
3-ketoacyl-ACP synthase 2	*KAS2*	52648	TGCTCAGTACGCCTACATCG	TTCTTCCACCGCTTGTTACC
3-ketoacyl-ACP synthase 3	*KAS3*	37367	AACGATCGCTTTACGCATTT	TGTGCCAGCTTGAGTGTTTC
fatty acyl-ACP thioesterase 1	*FAT1*	33198	CCGCGAACCATCGTTAGTAT	AAGTTCCCAACGGGCTAACT
fatty acyl-ACP thioesterase 2	*FAT2*	10454	TGTGCCGTATTGTCGTCCTA	GTAGAGCGGAAAGGCAGTTG
glycerol kinase	*GK*	50770	CCGCTGTAGGCATTACCAAT	TGCAAAGTAACTTGCCAACG
glycerol-3-phosphate acyltransferase	*GPAT*	54709	TTTTTGTGGCCGACTTATCC	CCTTGGTATCGACACGTCCT
plastidic lysophosphatidic acid acyltransferase	*ATS2a*	11916	GGCTTTCAAAATGGCCTACA	GGATCGTGGACGATGACTTT
plastidic lysophosphatidic acid acyltransferase	*ATS2b*	43099	TATTCGGCTTGGAAAGGATG	CCAGTCGGCGTTAATTTTGT
lysophosphatidic acid acyltransferase 3	*LPAAT3*	EG02461	AGGACGGTGCCTTTAAGGTT	TACCTTTCACGGGAATCGAC
lysophosphatidic acid acyltransferase 4	*LPAAT4*	45551	GCGACCAGTACCGAATTCAT	CCCAATTGTGAAGCCAATCT
phosphatidic acid phosphatase	*PAP*	40261	GTGGCGTTTGCCATAACTTT	TCAACGTCATGCCACAAAAT
carnitine o-acetyltransferase 2	*CAT2*	48078	CAATGTTGCCCGTGCCTAC	TTCCTGAAGCGTACTTGCCT
mitochondrial long chain acyl-CoA synthetase	*ACSL3*	54151	GGACGTGGAACTCTCCGTG	CGAACAAGCGTTTTTCCGCA
mitochondrial acyl-CoA dehydrogenase	*MACAD1*	11014	TTCCAAGACGGAAGAAATCG	GTTCCACAACATCGTGATCG
acyl-CoA dehydrogenase 1	*ACD1*	25932	GTGCTTCGGTTGGAGTCATT	CATTTCCTGGTTCCGAAAGA
acyl-CoA dehydrogenase 2	*ACD2*	20310	CGTCCACCTGTCCGATTACT	CTGACATGCGATACCAATGC
enoyl-CoA hydratase/3-hydroxyl-CoA dehydrogenas	*HADA*	35240	GATTGCGTCAAATTCGGTTT	GTGCTTTTTCATCTGCGTCA
enoyl-CoA hydratase	*ECH*	55192	GAATACGCCTACCGAACGAA	GATGACGCCCAGATTGATTT
3-hydroxyacyl-CoA dehydrogenase 2	*HAD2*	39681	CGGTGTTTGTCAAGTTGGTG	TTTTCCCCAAAGTCTCATCG
β-ketoacyl-CoA thiolase	*KCT3*	28068	ACCCACCTATTGTTCGCAAG	GGCAAAGGTTTGAGATTCCA
acetyl-CoA acetyltransferase	*KCT*	45947	GTCGATACCCTGCTCGAAAA	GCAAAGGCTTCGTTGATTTC
Histone H4	*H4*	26896	AGGTCCTTCGCGACAATATC	ACGGAATCACGAATGACGTT
